# Global Perspectives on Health Promotion Effectiveness

**Published:** 2008-06-15

**Authors:** Crystal Biruk

**Affiliations:** University of Pennsylvania, Philadelphia, Pennsylvania

**Figure:**
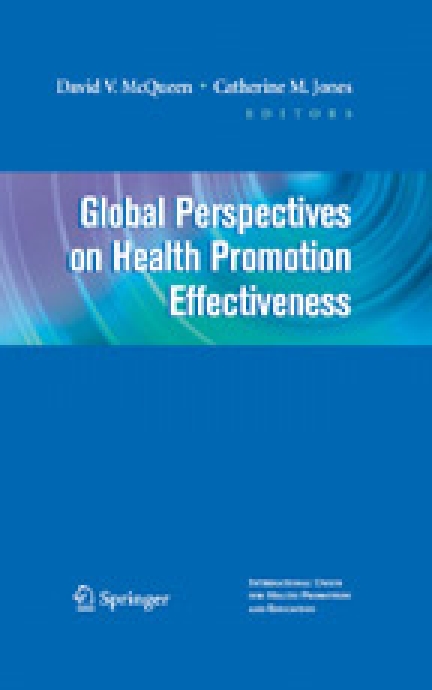


Targeted at a broad audience concerned with health promotion programs, *Global Perspectives on Health Promotion Effectiveness* includes contributions from a wide range of health promotion professionals involved in the Global Programme on Health Promotion Effectiveness of the International Union for Health Promotion and Education (IUHPE). The volume is divided into 4 sections, each unified by a common theme concerning evaluation and effectiveness in health promotion. Taken together, the contributors call for attention to the complexity of communities and settings where health interventions are staged (Dooris et al, Chapter 19), a more meaningful implementation of informed stakeholder input, and an expansion of the definition of legitimate evidence and measurement tools (Campostrini, Chapter 18). Along these lines, the volume successfully argues that the universally accepted concept of effectiveness must be reconsidered (especially De Salazar, Chapter 20, and Ridde et al, Chapter 22). *Global Perspectives on Health Promotion Effectiveness* questions the authoritative knowledge and evidence that have historically guided evaluation of health promotion endeavors.

Though a solid understanding of past efforts and mistakes in health promotion is essential to future successful interventions and evaluations, the volume falls short of providing the kind of ethnographic detail that would aid the larger community of health promotion in building on past collective experiences. The second section is ambitiously titled "Reports from the Field," but "the field" is conspicuously absent. That is, although the chapters report on specific interventions, the authors fail to provide ethnographic detail that could flesh out the field-based evidence they endorse and, thereby, permit future interventions to build on their knowledge. For example, although almost all authors in this section argue that multiple strategies across multiple settings are more likely to have a beneficial impact than single actions alone, no specific examples are cited to give substance to this approach. If effectiveness may be increased by "linking school based programs to out of school action" (St. Leger et al, Chapter 8), what do these links look like in real life, and how are they forged? Similarly, I think we can all agree with Howat et al that drinking behavior (abuse of alcohol) is best changed through "a combination of educational, organizational, economic and political actions," but I wonder how actual people link up with these multisectoral programs and experience them? In this way, the human faces are lost in health promotion models and discourse.

I appreciate the editors' early admission that the volume fails to include contributions from the developing world and fails to do justice to the complexity of the debate on evidence and effectiveness in the developing world. However, the glaring omission of case studies, debates, and contributions from these countries is still striking in a volume whose title incorporates the phrase "global perspectives." Although Jones and Mittelmark's chapter on the IUHPE blueprint for dialogue aims to explore how people with potentially different perspectives come together to collaborate as partners, it risks painting a too-rosy picture of a communication process that is often fraught, but masked by words like "partnership," "sharing," or "collaboration" (Metzler makes the insightful point that "common goals and incentives to collaborate are rare" [p. 239]). Early on, the editors point out that we often hear only about the successes of health promotion and never the failures; it seems that the authors of these essays also fail to stick their necks out and explore the specific challenges faced by global health promotion practitioners, either in their exchanges and collaborations with one another or in the field.

Some essays seem to valorize or romanticize civil society organizations (community-based organizations, nongovernmental organizations, faith-based organizations) as potentially more attuned to local realities, more democratic, less capital-centered, or more participatory (and thereby essential partners in health promotion activities). I caution against such a simplistic rendering as, certainly in sub-Saharan Africa, such organizations can be as mired in politics, corruption, and power struggles as governmental organizations, often with increasing financial resources at their disposal.

I did, however, appreciate the contributions that question the meaning of the term "community" in a globalized world (a word that fails to acknowledge the power relationships and diverse actors that form the much-lauded partnerships and networks that have been mainstreamed into global health-speak) (Labonte, Chapter 12). The chapter by Metzler is a most useful contribution: case studies of interventions in Kenya, India, and Colombia show that partnerships can be effective even in the absence of clear individual behavior change. McQueen's chapter successfully traces the theoretical debates and trajectories underlying health promotion, a task that is important for reflexive and critical health promotion practice.

Certainly, in a field with limited resources, attention to issues of evidence and effectiveness will continue to guide health promotion work. Likewise, improvements in accountability structures and evaluation mechanisms in a globalized world rely on meaningful strategies for cross-cultural collaboration. Although the first section of *Global Perspectives on Health Promotion Effectiveness* focuses on models for enhancing partnership and dialogue in these collaborations and on the functioning of global partnership, overall the volume only scratches the surface of the complexities of collaboration itself, despite collaboration being central to how evaluation is carried out and effectiveness is defined. Nevertheless, the volume does the discipline of health promotion a service by providing an opportunity to begin to rethink longstanding definitions of evidence and evaluation, and thus it moves health promotion toward practices that are more responsive to and inclusive of the concerns of the people on whom its efforts are focused.

